# Accouchements sur utérus cicatriciel en République Démocratique du Congo: épreuve utérine et facteurs déterminants de l’issue

**DOI:** 10.11604/pamj.2017.27.71.12499

**Published:** 2017-06-01

**Authors:** Félix Kitenge wa Momat, Pierre Akilimali Zalagile, Faustin Chenge Mukalenge, Oscar Numbi Luboya, Cléophas Tshibangu Kalala, Désiré Mashinda, Gilles Grangé, Olivier Mukuku, Fanny Malonga Kaj, Chamy Cham Lubamba, Joseph Bagambe Bwama, Célestin Kayembe Mukoko, Jean Baptiste Kakoma, Justin Kizonde Kalungwe

**Affiliations:** 1 Département de Gynécologie Obstétrique, Faculté de Médecine, Université de Lubumbashi, République Démocratique du Congo; 2 Ecole de Santé Publique, Faculté de Médecine, Université de Kinshasa, République Démocratique du Congo; 3 Département de Pédiatrie, Faculté de Médecine, Université de Lubumbashi, République Démocratique du Congo; 4 Département de Gynécologie Obstétrique, Pretoria, Afrique du Sud; 5 Département de Gynécologie Obstétrique, Hôpitaux Universitaires Paris Centre, France; 6 Département de Gynécologie Obstétrique, Cliniques Universitaires de Lubumbashi, République Démocratique du Congo; 7 Département de Gynécologie Obstétrique, Hôpital Général de Référence Sendwe, Lubumbashi, République Démocratique du Congo; 8 Département de Gynécologie Obstétrique, Hôpital de l’Amitié Sino-Congolais, Kinshasa, République Démocratique du Congo; 9 Département de Gynécologie Obstétrique, Hôpital Général de Référence de Bipemba, Mbuji-Mayi, République Démocratique du Congo

**Keywords:** Utérus cicatriciel, épreuve utérine, déterminants, score prédictif, Scarred uterus, trial of scar, risk factors, predictive score

## Abstract

**Introduction:**

L'objectif était d'identifier les principaux facteurs de risque associés à un échec d'épreuve utérine et définir un score prédictif d'accouchement sur utérus cicatriciel en République Démocratique du Congo.

**Méthodes:**

Étude multicentrique, transversale et analytique des patientes porteuses d'un utérus cicatriciel sur la période du 1^er^ janvier au 31 décembre 2013 dans quatre maternités de la République Démocratique du Congo (RDC). Un modèle de régression logistique a été construit pour identifier les facteurs associés à l'échec de l'épreuve utérine. De ce modèle, un score prédictif a été construit pour prédire l'échec de l'épreuve utérine dans les maternités de la RDC. La courbe ROC a été utilisée pour évaluer la capacité du score construit à identifier les patientes à risque de connaitre un échec de l'épreuve utérine. Le cut off point du score prédictif a été déterminé en fonction de la sensibilité et spécificité optimale via l'index de Youden. Tous les tests ont été réalisés au seuil de signification α=0,05.

**Résultats:**

Deux types de facteurs explicatifs de l'échec de l'épreuve utérine ont été retenus. Il s'agit d'un facteur sociodémographique (âge maternel) et de trois facteurs obstétricaux (hauteur utérine, présentation du fœtus et rupture prématurée des membranes). Le score de prédiction a été défini pour prédire l'échec de l'épreuve utérine. La construction de ce score s'est basée sur quatre éléments : l'âge maternel, l'état de la poche des eaux à l'admission, la hauteur utérine et la présentation fœtale. Le score minimal est de 4 et le score maximal est de 16. Le seuil est de 7. Un Score total supérieur ou égal à 7 traduit un risque d'échec de l'épreuve utérine.

**Conclusion:**

L'utilisation de ce score prédictif chez les patientes pourra améliorer la qualité dans les indications de la voie haute, l'augmentation des taux de césariennes prophylactiques ainsi que la meilleure sélection des patientes soumises à l'épreuve utérine. Ceci pourra également contribuer à la réduction de la morbi-mortalité fœto-maternelle liée à la gestion de l'accouchement sur utérus cicatriciels.

## Abstract (en)

**Objective:**

Our study aims to identify risks factors associated with failed trial of scar and to set a predictive score of labour in women with scarred uterus in the Democratic Republic of the Congo.

**Methods:**

We conducted a multicenter cross-sectional analytic study of patients with scarred uterus in four maternity units in the Democratic Republic of the Congo (DRC) from 1 January to 31 December 2013. Logistic regression model was used to identify factors associated with failed trial of scar. We set a predictive score based on this model in order to predict trial of scar failure in maternity units in the DRC. ROC curve was used to assess the ability of the set score to identify patients at risk for trial of scar failure. The cut off point for the predictive score was determined on the basis of the Youden-index-based optimal sensitivity and specificity. All the tests in our study were carried out by using a significance threshold of α=0.05.

**Results:**

Two explanatory factors in trial of scar failure were retained. They were the socio-demographic factor (maternal age) and three obstetric factors (fundal height, fetal presentation and premature rupture of membranes). Predictive score was set to predict trial of scar failure. This score was determined based on four elements: maternal age, evaluation of the gestational sac on admission, fundal height and fetal presentation. The minimum score was set at 4 and the maximum score was set at 16. The threshold value was set at 7. A total score greater than or equal to 7 reflects a risk of trial of scar failure.

**Conclusion:**

We set a predictive score to predict trial of scar failure. This score was determined based on four elements: maternal age, evaluation of the gestational sac on admission, fundal height and fetal presentation. A total score greater than or equal to 7 reflects a risk of trial of scar failure.

## Introduction

Le taux de césarienne a doublé en France et quadruplé aux Etats-Unis entre 1970 et 2010 [1]. Outre-Atlantique, la fréquence des cas de césarienne a sensiblement augmenté passant de 2>0,7% à 32,8% entre 1996 et 2007 [2]. L'accouchement sur utérus cicatriciel constitue un défi en obstétrique du fait d'absence d'unanimité dans la conduite à tenir. En France, près de 10% des parturientes ont un utérus cicatriciel [3]. Son étiologie principale demeure l'antécédent de césarienne et en cas d'accouchement sur utérus cicatriciel, la rupture utérine demeure la complication majeure qui survient le plus souvent pendant le travail [3]. Certains auteurs privilégient d'emblée la césarienne par crainte de la rupture utérine [4], tandis que d'autres préconisent un accouchement par voie basse si des paramètres précis sont observés [5]. Les sociétés savantes d'obstétrique dictent la conduite à tenir devant un utérus cicatriciel [6]. Les recommandations émises par l'American College of Obstetricians and Gynecologists (ACGO) [7], la Society of Obstetricians, Gynaecologists of Canada (SOGC) [8] et le Royal College of Obstetricians and Gynaecologists (RCOG) [9] insistent sur le fait que l'épreuve utérine est une option raisonnable. Cette épreuve utérine doit tenir compte du lieu d'accouchement, du plateau technique de la maternité, des caractéristiques de la patiente, de la qualification du personnel soignant, des facteurs obstétricaux et des pratiques obstétricales. Selon les études, elle varie de 28 à 70% [2,9]. Cependant la tentative d'accouchement par voie basse (TAVB) et la Césarienne Programmée Après Césarienne(CPAC) comportent aussi des risques pour le couple mère- enfant [3]. L'étude des facteurs de risque foeto-maternels des accouchements sur utérus cicatriciels (AUC) constitue une étape importante de l'amélioration des connaissances obstétricales sur les déterminants de la voie d'AUC. Pareille étude devrait discuter, en République Démocratique du Congo (RDC), l'association entre la voie haute, l'épreuve utérine, l'échec de l'épreuve utérine et la morbi-mortalité fœto-maternelle étant donné que les décisions de pratique clinique doivent prendre en compte ces différents paramètres. En effet, il existe une corrélation entre l'échec de l'épreuve utérine et la rupture utérine selon que ces deux éléments sont des facteurs synergiques de morbi-mortalité pour le couple mère-enfant. Bien plus, l'échec de l'épreuve utérine mal gérée majore le risque de rupture utérine avec mort fœtale et/ou maternelle.

En RDC, ni un score prédictif, ni des facteurs de risque des accouchements sur utérus cicatriciel n'ont été définis à ce jour. Peu d'études seulement qui mettent en évidence l'augmentation de l'incidence des accouchements sur utérus cicatriciels ont été menées. En 2013, aux Cliniques Universitaires de Kinshasa, la fréquence était de 8,45% avec une morbidité maternelle (rupture utérine) de 4,6%, une mortalité maternelle nulle et une mortalité périnatale de 29‰ [10], alors que cette fréquence était, trente années plutôt, de 2,43% dans la même institution avec une morbidité maternelle (rupture utérine) de 11,1%, une mortalité maternelle de 0,69‰et une mortalité périnatale de 90‰ [10]. Dans le but d'évaluer la prise en charge des AUC, notre étude a été menée dans quatre institutions hospitalières de trois milieux économiquement et socio-culturellement différents de la RDC (Kinshasa, Lubumbashi et Mbuji-Mayi). En vue de contribuer à la prévention et/ou à la minimisation du risque de morbi-mortalité maternelle en cas de parturition sur utérus cicatriciel dans un environnement obstétrical où le plateau technique et la qualification du personnel soignant font cruellement défaut comme dans certaines régions de la RDC. L'objectif de cette étude est d'identifier les principaux facteurs de risque associés à un échec de l'épreuve utérine afin de détecter les femmes à haut risque de césarienne au cours du travail. La connaissance de ces paramètres va permettre de définir un score prédictif des AUC en RDC. Le principe de prédiction du risque d'accouchement sur utérus cicatriciel est défini comme un processus clinique qui résulte de la conjugaison des éléments de l'anamnèse, des examens cliniques et paracliniques à l'établissement d'un meilleur pronostic f‰to-maternel pour les gestantes concernées.

## Méthodes

Il s'agissait d'une étude transversale multicentrique réalisée dans les 4 maternités de 4 hôpitaux de référence secondaire de la RDC, à savoir : Hôpital de l'Amitié Sino-Congolaise de Kinshasa (HASC), Hôpital Général de Référence de BIMPEBA (HGRB) à Mbuji-Mayi, Cliniques Universitaires de Lubumbashi (CUL) et Hôpital Général Provincial de Référence Sendwe de Lubumbashi. Ces hôpitaux reçoivent en effet les références et les évacuations sanitaires venant de certaines régions de la RDC. L'étude a couvert la période allant du 1^er^ janvier au 31 décembre 2013. Y ont été incluses, toutes les patientes avec antécédent de 1 à 2 césariennes (âge gestationnel ≥34 SA avec grossesse monofoetale et fœtus vivant), reçues pour une épreuve utérine ou une césarienne élective. Les présentations transverses et celles du front, franchement dystociques, ont été exclues de notre étude. Les sources d'informations étaient les registres d'accouchements, les comptes rendus opératoires, les partogrammes, les fiches de nouveau-nés en néonatologie et les fiches d'hospitalisation en post-partum. La fiche de récolte des données était remplie par huit enquêteurs recrutés parmi les prestataires. Les paramètres d'intérêt comprenaient : l'âge de la gestante (adolescente = âge <20 ans), le niveau d'instruction (peu instruite = sans instruction ou niveau primaire), le statut marital, la parité (primipare = 1, paucipare = 2, multipare = 3-4, grande multipare = ≥5), l'âge gestationnel, le nombre de césariennes antérieures, la notion d'accouchement par voie basse après le dernier accouchement par césarienne, l'intervalle entre les deux grossesses, la hauteur utérine, les contractions utérines, la présentation fœtale, la dilatation cervicale à l'admission, l'état des membranes fœtales, la durée du travail, le mode d'accouchement et les complications maternelles et périnatales ainsi que leurs issues vitales (décès ou survivants). La surveillance intrapartale était essentiellement clinique dans les 4 maternités utilisant notamment le stéthoscope de Pinard pour le suivi des bruits du cœur fœtal (BCF) et l'appréciation manuelle du régime contractile utérin. L'état du nouveau-né à la naissance a été apprécié par la mesure du score d'Apgar à la 5^ème^ minute. Au total 382 gestantes ont été admises durant la période d'étude dont 17 ont directement bénéficié d'une césarienne élective et ont été d'emblée exclues dans les analyses de la présente étude. En définitive, seules les 365 gestantes soumises à l'épreuve utérine spontanée (non déclenchée ni stimulée) ont été incluses dans cette étude. L'épreuve utérine, encore appelée « épreuve du travail sur utérus cicatriciel » ou « épreuve de la cicatrice » est la conduite d'un accouchement chez une patiente porteuse d'un utérus cicatriciel. Etait considéré comme échec de l'épreuve utérine, toute issue de la parturition sur utérus cicatriciel qui s'est soldé par une morbi-mortalité maternelle et périnatale et/ou tout échec de terminaison de l'accouchement par voie basse [3].

Les données ont été saisies sur ordinateur avec Epi Info 3.5.3 et le logiciel SPSS 21 a servi aux analyses. Les résultats ont été présentés sous forme de proportion pour les variables qualitatives avec leurs intervalles de confiance à 95%. Le test Khi-carré avec correction de Yates ou éventuellement le test exact de Fisher ont été utilisés pour la comparaison des petites fréquences. Les analyses bi-variées ont permis d'identifier les facteurs associes à l'échec de l'épreuve utérine. Un autre modèle de régression logistique a été construit pour identifier les déterminants de l'échec de l'épreuve utérine. Seules les variables ayant une valeur de p < 0,05 et moins susceptibles de subjectivité ont été introduites dans l'analyse multivariée. Pour réduire le biais probable sur la subjectivité dans les informations, les paramètres tels que le statut marital, l'antécédent d'avortement et le site d'accouchement n'ont pas été introduits dans l'analyse multivariée. Les odds ratio ajustés et leurs intervalles de confiance à 95% ont permis d'évaluer la force des différentes associations recherchées. De ce modèle, un score prédictif a été construit pour prédire l'échec de l'épreuve utérine dans les maternités de la RDC. A chaque modalité de la variable, un score a été attribué en fonction de son poids dans le modèle de régression logistique obtenu. La courbe ROC a été utilisée pour évaluer la capacité du score construit à identifier les gestantes à risque de connaitre un échec de l'épreuve utérine. Le seuil du score prédictif a été déterminé en fonction de la sensibilité et spécificité optimale via l'index de Youden. Tous les tests ont été réalisés au seuil de α=0,05. Du point de vue éthique, avant la récolte de données, les autorisations respectives de Médecins Directeurs des quatre Institutions concernées et du comité d'éthique de l'Université de Lubumbashi (N° approbation : UNILU/CEM/2015 du 15 février 2015) avaient été obtenues ainsi qu'un consentement éclairé, libre et verbal des parturientes. Les données étaient collectées de manière anonyme. L'étude n'a pas présenté de bénéfice direct pour les participants à l'étude.

## Résultats

### Caractéristiques sociodémographiques, cliniques et obstétricales des gestantes

Le Tableau 1 résume les caractéristiques des gestantes incluses dans l'étude en fonction de l'issue de l'épreuve utérine. Durant la période d'étude, 53% (n=193) des gestantes ont connu un échec de l'épreuve utérine parmi les 365 gestantes qui y ont été soumises. Comparativement aux deux autres groupes d'âge, 54% des gestantes de la tranche d'âge de 20 à 34 ans avaient connu un succès de l'épreuve utérine. L'HGRB avait un taux élevé de réussite de l'épreuve utérine comparativement aux trois autres maternités. De même, les gestantes vivant seules ont enregistré plus de réussites comparativement à celle en union. Les grandes multipares (>4) ainsi que les gestantes avec utérus surdistendu ont enregistré, quant à elles, un taux important d'échec lors de l'épreuve utérine. Une poche des eaux rompue et les présentations autres qu'occipitales étaient associées à des proportions élevées d'échec.

**Tableau 1:** Caractéristiques des gestantes et issues de l’épreuve utérine

Variable	n	Épreuve utérine		p
		Échec	Réussite	
**Âge**				
<20 ans	23	16(69,6)	7(30,4)	0,041
20-34 ans	211	98(46,4)	113(53,6)	*
>34 ans	131	78(59,5)	53(40,5)	0,019
**Site**				
CUL	63	37(58,7)	26(41,3)	0,04
HASC	103	60(58,3)	43(41,7)	0,02
Sendwe	73	41(56,2)	32(43,8)	0,07
HGR Bipemba	126	54(42,9)	72(57,1)	*
**Niveau d'instruction**				
Faible	172	98(57,0)	74(43,0)	0,114
Bonne	193	94(48,7)	99(51,3)	*
Statut marital				
En union	265	153(57,7)	112(42,3)	0,001
Vivant seule	100	39(39,0)	61(61,0)	*
**Parité**				
< 4	170	78(45,9)	92(54,1)	*
≥ 4	195	114(58,5)	81(41,5)	0,017
**Antécédent d'avortement**				
Non	318	153(48,1)	165(51,9)	*
Oui	47	39(83,0)	8(17,0)	< 0,001
**Espace inter génésique**				
>24 mois	45	20(44,4)	25(55,6)	*
≤24 mois	320	172(53,8)	148(46,2)	0,244
**Poche des eaux**				
Rompue	116	89(76,7)	27(23,3)	<0,001
Intacte	215	88(40,9)	127(59,1)	*
**Présentation**				
Céphalique	254	106(41,7)	148(58,3)	*
Autres	111	86(77,5)	25(22,5)	<0,001
**Hauteur utérine**				
<30 cm	278	127(45,7)	151(54,3)	*
30–34 cm	45	33(73,3)	12(26,7)	0,001
>34 cm	33	23(69,7)	10(30,3)	0,011

* : niveau de référence

### Issue de l'épreuve utérine

Le Tableau 2 rapporte que comparativement à l'HGRB, plus de la moitié des patientes ont accouché par voie vaginale dans les trois autres sites (p=0,001).

**Tableau 2:** Issue de l'épreuve utérine par site

Variables	HASC	CUL	p*	HPR Sendwe	p*	HGRB	p*
	**n=112**	**n=64**		**n=78**		**n=128**	
**EVB n=365 (95,55)**							
Succès EVB n = 183(50,14)	45 (43,69%)	28 (44,44%)		32 (43,84%)		78 (62,00%)	
Échec EVB n=182(49,86)	58 (56,31%)	35 (55,56%)	0,924	41 (56,16%)	0,984	48 (38,00%)	1,000

HASC: Hôpital de l’Amitié Sino-Congolais ; HGRB: Hôpital Général de Référence de Bipemba ; CUL: Cliniques Universitaires de Lubumbashi ; EBV: épreuve d’accouchement par voie basse

### Facteurs associés à l'échec de l'épreuve utérine

Le Tableau 3 présente les facteurs associés à l'échec de l'épreuve utérine. Les gestantes âgées de <20 ans (ORa=3,32 [1,11-9,87]), une poche des eaux rompue (ORa=3,95 [2,26-6,90]), une présentation dystocique (ORa=5,86 [3,13-10,95]) et un utérus surdistendu (ORa=2,73 [1,13-6,57]) étaient associés de façon significative au risque de l'échec de l'épreuve utérine.

**Tableau 3:** Facteurs associés à l’échec de l’épreuve utérine en analyse multivariée

Variables	OR brut [IC95%]	p	OR ajusté [IC95%]	p
**Age**				
<20 ans	2,64 [1,04-6,67]	0,041	3,32 [1,11-9,87]	0,031
20-34 ans	1,00		1,00	
>34 ans	1,70 [1,10-2,64]	0,019	1,58 [0,89-2,80]	0,119
**Parité**				
< 4	1,00		1,00	
≥ 4	1,66 [1,10-2,51]	0,017	1,43 [0,82-2,52]	0,211
**Poche des eaux**				
Rompue	4,76 [2,86-7,92]	<0,001	3,95 [2,26-6,90]	< 0,001
Intacte	1,00		1,00	
**Présentation**				
Céphalique	1,00		1,00	
Autres	4,80 [2,88-8,00]	< 0,001	5,86 [3,13-10,95]	< 0,001
**Espace inter génésique**				
>24 mois	1,00			
≤24 mois	1,45 [0,78-2,72]	0,244		
**Hauteur utérine**				
<30 cm	1,00		1,00	
30-34 cm	3,27 [1,62-6,59]	0,001	2,75 [1,26-6,00]	0,011
>34 cm	2,74 [1,26-5,96]	0,011	2,73 [1,13-6,57]	0,025

### Philosophie pour la prédiction de l'issue d'une épreuve utérine : motivation, fiabilité, rentabilité et confection du score prédictif des AUC

### Méthodologie de la confection du score

Un score a été établi partant du modèle de régression logistique du Tableau 3 ne reprenant que les facteurs statistiquement significatifs. Le score minimal était de 4 et le score maximal de 16. Le seuil était de 7. Un score total supérieur ou égal à 7 traduisait un risque d'échec de l'épreuve utérine. Tableau 3 présente les critères de l'établissement du score et le Tableau 4 donne les éléments de validité de ce score.

**Tableau 4:** Critères d’établissement du score de prédiction des AUC

Critères d'établissement du Score	Score
**Âge**	
< 20 ans	3
20-34 ans	1
>34 ans	1
**Présentation**	
Céphalique non dystocique	1
Autre	6
**Poche des eaux**	
Rompue	4
Intacte	1
**Hauteur utérine**	
< 30 cm	1
≥ 30	3

### Validation et interprétation du score de prédiction des AUC

Le score de prédiction des AUC permet de classifier correctement 72,7% des épreuves utérines avec une sensibilité de 71,6%, une spécificité de 74,0%, une valeur prédictive positive de 75,1% et une valeur prédictive négative de 70,4% Tableau 5

**Tableau 5:** Validité du score de prédiction des AUC

Score de prédiction des AUC	Réponse de l’épreuve utérine observée		Total
	**Échec**	**Réussite**	
≥ 7	121	40	161
< 7	48	114	162
**Total**	169	154	323

## Discussion

### Facteurs associés à l'échec de l'épreuve utérine

Les facteurs associés à l'échec de l'épreuve utérine permettent à l'obstétricien de renoncer à la possibilité d'accouchement par voie naturelle. Le taux d'échec de l'épreuve utérine est variable dans la littérature et est compris entre 13 et 51% [2,3]. Les 67 études (14 prospectives et 53 rétrospectives) de la méta-analyse de Guise et al., montrent que le taux de succès de l'épreuve utérine était de 74% (IC 95% [72-75], n =368 304) avec une hétérogénéité importante (I2 >98%) sans que soient retrouvés de facteurs explicatifs à cette différence (année d'étude, pays d'origine de l'étude, terme d'accouchement et type d'étude) [2]. Dans notre étude, quatre facteurs explicatifs de l'échec de l'épreuve utérine ont été retenus, à savoir : l'âge maternel, la hauteur utérine, la présentation du fœtus et la rupture prématurée des membranes.

### Âge maternel et échec de l'épreuve utérine

Existe-t-il un seuil d'âge maternel dans notre milieu au-dessus ou en dessous duquel, en raison d'un taux de succès de l'épreuve utérine trop bas et/ou de complication materno-périnatale élevée, une césarienne serait préférable à une épreuve utérine? Dans notre série, les gestantes âgées de moins de 20 ans (ORa=3,32 [1,11-9,87]) étaient statistiquement plus exposées à connaître un échec de l'épreuve utérine. Huit études de cohorte ont rapporté, en analyse uni ou multivariée, un lien entre âge maternel et le taux de succès de l'épreuve utérine. L'on a noté un taux de succès plus élevé parmi les patientes de moins de 40 ans dans cinq de ces études [7, 11–14] alors que trois études n'ont pas retrouvé de différence significative sur le taux de succès de l'épreuve utérine en fonction de l'âge maternel [13, 15]. Deux études se sont intéressées à l'effet de l'âge maternel sur le risque de rupture utérine et sur le taux de succès de l'épreuve utérine [16,17]. Toutes ces deux études concluent à une réduction de possibilité d'accoucher par les voies naturelles avec l'augmentation de l'âge maternel. Ce résultat n'est pas spécifique chez les patientes avec utérus cicatriciel puisqu'il est aussi retrouvé chez les patientes avec utérus indemne de cicatrice [18]. Dans l'étude de Bujold et al. [16], les taux de succès de l'épreuve utérine chez les patientes n'ayant jamais accouché par les voies naturelles étaient respectivement de 71,9%, 70,7% et 65,1% (p=0,06), chez celles âgées de moins de 30 ans, de 30 à 35 ans et de plus de 35 ans ; tandis qu'ils étaient respectivement de 91,5%, 91,1% et de 82,9% (p=0,005) chez les patientes ayant déjà accouché par les voies naturelles. En tout état de cause, la littérature ne fournit pas d'éléments suffisants pour déterminer un seuil d'âge maternel au-delà duquel une césarienne élective est préférable à l'épreuve utérine en cas d'accouchement sur utérus cicatriciel. Toutefois, l'avenir obstétrical de la gestante est un élément à intégrer à l'information et à la décision des modalités d'accouchement chez ces patientes. Dès lors, il serait excessif de conclure que l'âge de la patiente à lui seul serait associé à la réussite et/ou à l'échec de l'épreuve utérine. Notre étude a, par contre, démontré que les patientes de moins de 20 ans avaient 6 fois plus de risque de connaître l'échec de l'épreuve utérine comparativement à celles plus âgées.

### Présentation dystocique et échec de l'épreuve utérine

Dans l'espèce humaine, la disproportion céphalo-pelvienne oblige la tête fœtale à se fléchir pour l'accouchement, afin de réduire son diamètre antéropostérieur. Dans moins de 1% des cas, ce mécanisme ne se produit pas et c'est au contraire une déflexion plus ou moins importante de la tête que l'on observe; ce qui définit les présentations céphaliques défléchies. Ces présentations sont caractérisées par un travail qui est long et une mécanique obstétricale complexe. Les variétés postérieures de la présentation du sommet paraissent classiquement moins eutociques que les variétés antérieures avec un pronostic obstétrical et fœtal moins favorable [19]. L'attitude communément admise devant l'association d'un utérus cicatriciel et d'une présentation autre que céphalique bien fléchie est la réalisation d'une césarienne itérative systématique [20]. Cependant, cette attitude n'est pas acceptée par certains auteurs qui rapportent que l'épreuve utérine dans une présentation de siège donne des bons résultats, avec de faibles taux de complications [21]. Le problème de l'accouchement par voie vaginale d'un fœtus en présentation de siège est le risque de rupture utérine lors des manœuvres d'extraction. Par crainte du risque de rupture utérine lié notamment aux manœuvres d'extraction du siège sur utérus cicatriciel, certains auteurs optent d'emblée pour une césarienne itérative systématique devant l'association d'un utérus cicatriciel et d'une présentation de siège [22, 23]. Cependant, certains auteurs rapportent un faible taux de complications en cas de réalisation d'une épreuve utérine sur présentation de siège [24]. Dans notre série, les patientes avec une présentation dystocique étaient exposées au risque de connaître l'échec au cours de l'épreuve utérine (ORa=5,86 [3,13-10,95]). Ceci peut avoir un lien avec la genèse des présentations dystociques, dont les étiologies diffèrent pour chacune d'entre elles [21]: présentations du front et bregmatique : bassins suspects, malformations fœtales, hydramnios et anomalies du cordon; Présentation de face : multiparité, macrosomie, gémellité et poids <2500 grammes sont les facteurs. En conséquence, la mise en évidence d'une présentation dystocique au cours du 3ème trimestre de la gestation implique la multiplication des consultations et la réalisation d'une enquête étiologique de cette présentation avant de conclure à une origine idiopathique.

### Macrosomie et échec de l'épreuve utérine

Dans notre série, les gestantes porteuses d'un utérus surdistendu étaient associées de façon significative à un risque d'échec de l'épreuve utérine (ORa=2,73 [1,13-6,57]). Etant un paramètre anténatal, dans la méthodologie utilisée, le poids fœtal a été exclu dans le modèle de régression logistique principalement pour deux raisons : d'abord, le poids fœtal est associé naturellement à la hauteur utérine (fiabilité subjective parce qu'opérateur dépendant), ceci peut entraîner l'instabilité du modèle suite à la colinéarité. Ensuite, la prédiction de l'échec ou du succès de l'épreuve utérine avec le paramètre pondéral du nouveau-né suppose que celui-ci soit déjà né (à moins de se référer au poids échographique en assumant ses limites). Dans ce cas, la hauteur utérine devient une variable proxy qui mesure indirectement aussi le poids fœtal qui offre malgré tout un avantage pratique et économique (l'échographie n'étant pas à la portée de tout le monde). La probabilité d'accouchement par voie basse sur utérus cicatriciel est en relation directe avec le poids fœtal [25]. La macrosomie fœtale est définie par un poids de naissance supérieur à 4000 grammes et/ou par un poids de naissance supérieur au 90ème percentile d'une courbe de référence de la population donnée. Cependant, l'estimation du poids fœtal par mesure échographique reste très imprécise avec un risque d'environ 50% de faux positifs pour prédire un poids de naissance supérieur à 4000 grammes [26]. La survenue d'une macrosomie fœtale chez une patiente antérieurement césarisée est une situation fréquente, représentant environ 16% des accouchements sur utérus cicatriciel [27]. Le taux de succès de l'épreuve utérine chez les patientes ayant donné naissance à des macrosomes varie dans la littérature entre 40 et 92% avec une moyenne de 69% dans une revue de la littérature compilant 807 cas [28]. Flamm rapportait un taux de succès de 78% pour le groupe ayant un poids de naissance <4000 grammes, de 58% pour le groupe ayant un poids compris entre 4 000 et 4 499 grammes, et de 43% seulement si le poids de naissance est supérieur à 4 500 grammes. Comparativement à l'épreuve de travail sur utérus non cicatriciel, Flamm notait aussi que la différence du taux d'accouchement par voie basse des macrosomes avec les patientes antérieurement césarisées était statistiquement significative (90% vs 55%) [29]. Le risque de rupture utérine lors de l'épreuve utérine en cas de macrosomie foetale est estimé à 0,3% dans la série de Flamm, incluant 301 cas, et à 0,7% dans la série de Phelan incluant 140 cas. Ce risque semble similaire à celui observé en cas de poids fœtal normal (< 4 000 grammes). Concernant le risque de déhiscence utérine, il est de l'ordre de 0,7% dans la série de Flamm et de 2,1% pour Phelan. Ce risque n'est non plus différent de celui retrouvé pour un poids fœtal inférieur à 4 000 grammes [25, 29] La tentative d'accouchement par voie basse en cas de macrosomie fœtale présente un risque majeur de dystocie des épaules, exposant à un risque de traumatisme obstétrical, en particulier, la paralysie du plexus brachial [30]. Mais la majorité des auteurs estiment qu'en absence d'autres facteurs de risque, en particulier un diabète maternel, une suspicion de macrosomie fœtale ne justifie pas la pratique systématique de la césarienne, d'autant plus que les techniques actuelles d'estimation du poids fœtal ne sont pas assez fiables [31]. La césarienne secondaire faite après échec de l'épreuve utérine comporte plus de morbidité maternelle, en particulier des complications graves [32]. Ainsi, une évaluation minutieuse clinique, et parfois paraclinique, des conditions obstétricales avant toute épreuve utérine est d'une importance capitale permettant d'exclure les mauvaises confrontations fœto-pelviennes et d'éviter une épreuve utérine vouée à l'échec avec ses complications éventuelles. Une fois l'épreuve utérine acceptée, une prise en charge active du travail sous surveillance stricte en présence d'une équipe obstétricale entraînée est indispensable. L'établissement du partogramme doit être de rigueur afin de dépister et traiter à temps toute anomalie du travail. Une stagnation de la dilatation en phase active du travail au-delà de 2 heures après correction des anomalies dynamiques doit faire interrompre l'épreuve utérine et pratiquer une césarienne; de même pour l'absence d'engagement de la tête foetale après une heure de dilatation complète.

### État des membranes et échec de l'épreuve utérine

La rupture prématurée des membranes à terme est définie par une perte de liquide amniotique suite à une ouverture spontanée de l'œuf après 37 SA sans mise en travail de la gestante [33]. Pour Savitz [34], par exemple, l'incidence peut varier de 31% si aucun délai de mis en travail spontanée n'est pris en compte, à 6% lorsqu'il passe à 12 heures. En obstétrique, la rupture prématurée des membranes est une pathologie très fréquente. Elle complique 3 à 10% des grossesses dont 70 à 80% à terme [35]. Certains auteurs évoquent un risque relatif de travail hypercinétique en tout début de travail car les taux des prostaglandines dans le liquide amniotique sont toujours supérieurs aux taux maternels à raison de 24 à 85 pg pour les prostaglandines E et de 5 à 10 pg pour les prostaglandines F. Le liquide amniotique contient des prostaglandines (PGE2 et PGF2) qui ont une action collagénolytique engendrant la maturation du col par relâchement de sa trame collagénique. En parallèle, elles augmentent le nombre de récepteurs utérins aux ocytociques et potentialisent leur effet utérotonique. La libération de prostaglandines semble donc affaiblie en cas de rupture d'une poche amnio-choriale. Cet amoindrissement peut-il être responsable d'une atténuation des mécanismes biochimiques normalement engendrés et donc responsables d'un allongement de la phase de latence ou d'un retard dans l'amélioration des conditions locales (score de Bishop?) [33, 36]. Dans notre série, une poche des eaux rompue était statistiquement associée à l'échec de l'épreuve utérine (OR=3,95 [2,26-6,90]). En considérant d'une part que le taux élevé des prostaglandines induisent des contractions plus violentes (facteur de risque de la rupture utérine chez une gestante porteuse d'un utérus cicatriciel) [37], et d'autre part le fait que le risque de ralentissement du travail est aussi plus élevé en cas de rupture prématurée des membranes (la poche des eaux ne protégeant plus la tête fœtale), celle-ci subit directement les variations de pression intra-ovulaire induites lors des contractions. Les cas d'échec de l'épreuve utérine dans notre série ne seraient-ils pas dûs à ce mécanisme?

### Score de prédiction des AUC

Les scores prédictifs ont pour but d'évaluer d'une part la probabilité de succès de l'épreuve utérine [38], et d'autre part, la probabilité de l'échec de l'épreuve utérine [39]. Dans leur étude rétrospective de 10828 utérus cicatriciels, Bensaid et al. ont élaboré un score prédictif de succès de l'épreuve utérine qui a bénéficié d'une validation interne [40]. Seuls trois facteurs étaient associés à la réussite de l'épreuve utérine (absence de cause récurrente de césarienne, absence d'antécédent de macrosomie fœtale et absence d'anémie maternelle). Ce score établit une cotation de 1 lorsque le facteur était absent et de 0 lorsqu'il était présent. D'autres auteurs ont tenté d'établir un score anténatal prédictif d'échec de l'épreuve utérine dans une étude cas-témoins de 336 sujets. Cette étude a permis de prédire un taux d'échec de l'épreuve utérine dans 63% des cas bien que certains auteurs récusent la fiabilité de ce score [41]. À partir d'une cohorte prospective de 5000 patientes, dans la série de Flamm et al. [42], cinq facteurs ont été retenus pour la prédiction du taux de succès de l'épreuve utérine (âge maternel, antécédent d'accouchement par voie basse, indication de la césarienne antérieure, toucher vaginal à l'admission, dilatation et degré d'effacement cervical), une corrélation positive était retrouvée entre le taux de succès de l'épreuve utérine et le score calculé compris entre 49,1% pour les scores 0-2 et 94,9% pour les scores 8-10. Notre score a été défini pour prédire l'échec de l'épreuve utérine. La construction de ce score s'est basée sur quatre éléments : l'âge maternel, l'état de la poche des eaux à l'admission, la hauteur utérine et la présentation fœtale. Le score minimal est de 4 et le score maximal est de 16 avec un seuil de 7. Un score total supérieur ou égal à 7 traduit un risque d'échec de l'épreuve utérine. Etant donné que la majorité des scores rapportés dans la littérature sont construits soit à partir d'effectifs trop faibles, de cohortes rétrospectives ou de cohortes trop anciennes aux pratiques médicales datées, ce qui introduit de nombreux biais. Notre score a l'avantage d'avoir permis de valider une méthodologie assez robuste (étude prospective, régression logistique multiple, cross-validation, validation interne). L'aire sous la courbe ROC de 74% rend sa fiabilité acceptable. D'où nous avons convenu de le nommer « Score de MOMAT ».

### Discussion méthodologique

En effet, il s'agissait d'une étude transversale multicentrique dont les quatre maternités n'étaient pas sélectionnées de manière aléatoire et donc pas représentatives de l'ensemble des maternités de la RDC. Cette manière de sélectionner les maternités suggère un biais de sélection. Certaines informations telles que les antécédents d'avortement, le statut marital, la parité et le nombre de césarienne antérieure ont été rapportés par les gestantes. Cette technique utilisée pour collecter ces données nous exposent à des biais de mauvaise classification (biais d'information). Les erreurs de classification (manque de validité intrinsèque) réduisent la capacité de mettre en évidence l'effet réel des facteurs de risque sur le phénomène étudié, en sous-estimant l'odds ratio (biais directionnel vers l'unité ou dilution de l'effet). Ces erreurs peuvent survenir si les questions sont mal formulées dans le questionnaire. Le soin apporté dans la conception et la validation du questionnaire ainsi que le suivi très régulier des enquêteurs sur le terrain a permis de réduire de telles erreurs. Concernant les éventuels facteurs de confusion, nous avons restreint, dans la conception de cette étude, la participation à l'étude des gestantes avec 1 ou 2 antécédents de césarienne. Nous avons par la suite utilisé dans les analyses statistiques les techniques multivariées et aucune confusion n'a été constatée après ajustement des modèles estimés. Mais il peut subsister une confusion résiduelle par le fait que toutes les variables censées expliquer l'issue de l'épreuve utérine n'ont pas été introduites dans le modèle. La confusion résiduelle peut résulter aussi de la manière dont nous avons catégorisé certaines variables continues tels que l'âge et la parité de la gestante par exemple. Concernant la précision des résultats de cette étude, nous avons utilisé une taille suffisamment grande pour garantir une bonne précision avec une puissance statistique acceptable. En effet, la précision des résultats dépend de la taille d'échantillon, de la variabilité des paramètres étudiés (IC à 95% des mesures de fréquence et d'effet) et de l'absence ou de la présence d'erreurs aléatoires. Les trois principales sources d'erreurs aléatoires sont les erreurs d'échantillonnage, les erreurs de mesure ou erreurs de classification et les variations biologiques individuelles. Une étude transversale ne permet pas de faire une analyse des tendances qui aurait été d'une plus-value remarquable. Il aurait été souhaitable d'avoir un site d'étude à l'Est de la RDC pour prétendre avoir couvert tout le pays. Mais cela n'a pas été possible en raison notamment des contraintes opérationnelles. Comme dit ci-haut, pour simplifier les procédures de recrutement, nous avons seulement inclus les gestantes des 4 maternités qui prennent en charge un grand nombre des gestantes avec utérus cicatriciel. Ces maternités attirent des gestantes de différentes communes de ces différentes villes. Ces quatre maternités et la population qui les fréquentent ne sont pas probablement différentes de celles qui ne sont pas incluses dans l'étude. Nous pensons humblement donc que les résultats de cette étude peuvent raisonnablement être extrapolés sur l'ensemble du pays en assumant qu'il n'y pas de différence biotypique majeure entre les patientes de Kinshasa, de Mbuji-Mayi et de Lubumbashi et celles de partout ailleurs en RDC. Malgré les limites signalées ci-haut, cette étude a le mérite d'être la première à proposer un score identifiant l'issue pour une épreuve utérine dans notre milieu.

## Conclusion

Les facteurs de succès et d'échec d'une épreuve utérine sont des informations capitales qui contribuent efficacement à la décision pour le praticien et la patiente concernant les modalités d'accouchement sur utérus cicatriciel. Deux types de facteurs explicatifs de l'échec de l'épreuve utérine ont été retenus. Il s'agit d'un facteur sociodémographique (âge maternel) et de trois facteurs obstétricaux (hauteur utérine, présentation du fœtus et rupture prématurée des membranes). Le score de MOMAT a été défini pour prédire l'échec de l'épreuve utérine. La construction de ce score s'est basée sur quatre éléments : l'âge maternel, l'état de la poche des eaux à l'admission, la hauteur utérine et la présentation fœtale. Le score minimal est de 4 et le score maximal de 16. Le seuil point est de 7. Un score total supérieur ou égal à 7 traduit un risque d'échec de l'épreuve utérine. L'utilisation de ce score prédictif chez les patientes pourra améliorer la qualité dans les indications de la voie haute, l'augmentation des taux de césariennes prophylactiques ainsi que la meilleure sélection des patientes soumises à l'épreuve utérine. Ceci pourra également contribuer à la réduction de la morbi-mortalité fœto-maternelle liée à la gestion de l'accouchement sur utérus cicatriciels.

### Etat des connaissances actuelle sur le sujet

- La recrudescence de la césarienne dans les pays développés s'est accompagnée d'un bénéfice proportionnel pour le couple mère-enfant;
- En Afrique subsaharienne, l'accouchement sur utérus cicatriciel constitue un défi en obstétrique du fait d'absence d'unanimité dans la conduite à tenir;
- Aucune donnée récente disponible sur ce sujet en République Démocratique du Congo.

### Contribution de notre étude à la connaissance

- Première étude multicentrique en République Démocratique du Congo;
- L'étude proposée est la première étude analytique globale permettant d'établir un score pour prédire l'échec de l'épreuve utérine. La construction de ce score s'est basée sur quatre éléments: l'âge maternel, l'état de la poche des eaux à l'admission, la hauteur utérine et la présentation fœtale.

## Conflits d’intérêts

Les auteurs ne déclarent aucun conflit d'intérêts.

## Remerciements

Les auteurs tiennent à remercier les médecins directeurs et les infirmières responsables de maternités des hôpitaux concernés pour leur appui dans la collecte de données.
